# Proceedings of the American Society of Dental Surgeons

**Published:** 1854-01

**Authors:** Charles Bonsall


### *Proceedings oj the American Society of Dental Surgeons.*

[The following report of the proceedings of this Society was handed in by Dr. Bonsall for publication in the last number of the Register, and we regret very much that it was overlooked, and particularly so, as we had requested the Dr. to prepare an abstract of the Society's proceedings for publication, and also because the Society adjourned to meet in this city, on the first Tuesday in August, 1854. We hope the profession in the West will bear this in mind, and that many will attend. We can assure those who are not members that they will be greeted as brethren, and the Society be glad to have them present. We hope that many of our brethren of the East will take occasion to visit the "Queen City," and thus see our fertile lands and our "log cabins with the latch-string out."]

*To Dr. Jas. Taylor, Editor of the Register:*

Having been disappointed several times in my intention of attending the sittings of the American Society of Dental Surgeons, of which I had been a member for nine or ten years, I this year arranged my business so as to be present, and, not finding you there, concluded to make a few notes for the Register; and, though meagre and imperfect, you are welcome to make use of them, if you desire. One thing in the business particularly was very gratifying to me, and that was the resolution to hold the next annual meeting in Cincinnati; and I may here hope that our Eastern friends will meet with a hearty welcome from the profession here next August.

I would remark, before commencing, that the place of meeting this year, West Point, N. Y., is much more beautiful and interesting than I had supposed, judging from seeing it from the river only, as I had before done, and that Cozzens' Hotel, at which most of our meetings were held, and where we generally stayed, is one of the best hotels in the country; and a ramble up the mountain or over to the United States grounds, to see the morning drill of the Cadets, was a healthful and pleasant beginning for the business of the day.

*Proceedings.*—The Society met at Ryder's Hotel, on Tuesday morning, August 2, Dr. Eleazar Parmly, of New York, President, in the chair. There being but a small attendance, adjourned, to meet to-morrow morning, at 9 o'clock.

Wednesday morning.—There was a pretty good attendance of members, together with a number of dentists, not members, but taking an interest in our proceedings. Dr. E. Townsend offered the usual resolution that all the dentists at the Point be invited to be present, and a committee was appointed to ask them to join with us.

The Treasurer's report was read, and audited with a balance of $146.86 in the treasury.

Committee on Microscopic Observations reported progress, and were continued.

Committee on Foreign Dental Literature were continued; no report.

The case of Dr. Dwinelle now coming up, was disposed of, after discussion, by carrying into effect the resolution passed two years ago, suspending him from membership.

Dr. E. Townsend now read an essay on "Dental Patents," condemning strongly the practice of obtaining them by members of the profession, and showing a strong contrast between *copy rights* and *patent rights.*

The report of the Committee to revise the Constitution was read by Dr. J. D. White, the chairman, and, after being considered by sections and amended, was unanimously adopted.

The by-laws next coming under consideration, one article, on dental ethics, occasioned a good deal of discussion. It was as follows:

"Art. 13. Any dentist who shall procure a patent for a remedy or instrument of dental surgery, or who deals in patent remedies or nostrums, shall be disqualified for membership."

One or two members contended that there should be some qualification, by which such members as had expended time and labor in bringing out any important improvement might be allowed to patent them, or in some way receive compensation. Most of the members gave their views, after which the article was adopted almost unanimously, as read.

Drs. J. D. White and E. Townsend were appointed a committee to print the revised constitution and by-laws.

On motion, adjourned to meet at Cozzens' Hotel at 4½ o'clock, P. M.

Afternoon Session.—Dr. Dunning brought to the notice of the Society a pamphlet circular, intended as a *strong* advertisement, and issued by a member of the Society. It was read, and the manner in which the author makes known to the public his connection with the Society was deemed highly disrespectful and unwarranted, and demanding some action by way of rebuke. It was, on motion,

*Resolved*, That the following resolution be adopted as a by-law of the Association:

*Resolved*, That any member of this Society who shall extol his own peculiar merits over a fellow-practitioner in the public prints, or employs means of advertisement which may be regarded by this Society as lowering its dignity or compromising its character, shall be impeached, suspended or expelled.

On motion, the Secretary was directed to transmit to Dr. Thomas Palmer a copy of the constitution and by-laws.

A misunderstanding having occurred between Dr. C. A. Harris and the committee appointed last year to confer with him on the subject, in relation to the payment of a bill for printing tabular sheet, after a full examination, the Society endorsed the action of the committee, and passed a resolution condemnatory of the action of Dr. H. in the transaction.

Dr. Hawes inquired whether the 13th article of the by-laws affected a member *using* a patent instrument. It was decided that it did not.

Adjourned till 9 A. M., to-morrow.

Thursday morning, Aug. 4th.—The Society went into an election for officers, which resulted as follows:

President—Dr. Elisha Townsend. Vice Presidents—Drs.

J. H. Foster, J. Tucker, W. H. Goddard. Corresponding and Recording Secretary—Dr. David R. Parmly. Treasurer, Dr. E. J. Dunning. Librarian—Dr. D. R. Parmly. Publishing Committee—Drs. I. D. White, E. Townsend, D. R. Parmly. Examining Committee—Drs. E. Townsend, J. D. White, C. C. Williams, J. Tucker, and A. C. Hawes. Committee on Inventions and Improvements—Drs. J. H. Foster, E. G. Tucker, J. Parmly, and J. D. White.

Dr. Jahiel Parmly was appointed to deliver the opening address, and the following members to read essays at the next annual meeting: Drs. Robertson, A. C. Hawes, C. C. Williams, A. Westcott, and E. G. Tucker.

A vote of thanks was passed to Dr. E. Townsend, for his very able address, and a similar vote to Dr. E. Parmly, for the courteous manner in which he had presided over the meetings for several years; also a resolution of thanks to the Messrs. Cozzens, proprietors of the hotel, for the obliging and accommodating spirit which they had manifested toward the Association.

Adjourned till 4½ o'clock, P. M.

Afternoon Session.—The Examining Committee having reported the names of the following gentlemen for membership, they were ballotted for, and unanimously elected, viz: Dwight Tracy, M. D., D. D. S., Willimantie, Conn.; J. S. Clark, D. D. S., New Orleans, La.; Francis Field, dentist, Waltham, Mass.; R. P. Berry, Newport, Rhode Island; after which the Society adjourned, to meet in Cincinnati, Ohio, on the first Tuesday in August, 1854.

I may say, in closing, that the meeting was well attended, and that a great deal of good feeling prevailed, and I believe that the general impression was that, under the *revised constitution*, and with present feelings, the Society would continue with greater prospect of usefulness than ever. It was to be regretted that, owing to an error in the published proceedings of last year, a number of members, who had intended to be with us, did not get there in time—among others, Dr. Evans, of Paris, who had arranged his visit to this country with a view of attending the meeting. There is, also, one feature of our Mississippi Valley Association, which might be engrafted into the plan of business of the Society to advantage. I allude to the interlocutory meetings we have held of evenings, etc.

Yours sincerely,

Charles Bonsall.

